# Supplementary material for: Quality and credibility of clinical practice guidelines recommendations for the management of neonatal hypoglycemia. A protocol for a systematic review and recommendations’ synthesis
Source: PLoS One. 2023 Jan 20;18(1):e0280597. doi: 10.1371/journal.pone.0280597 (PMC9858031; doi:10.1371/journal.pone.0280597)
Supplement: S1 File — (DOCX) [file pone.0280597.s002.docx]

**Supporting information file**

**Preliminary Ovid MEDLINE(R) Search Strategy**

1. infan$.tw.
2. exp Infant, Newborn/ or exp Infant, Newborn, Diseases/ or exp Infant, Small for Gestational Age/ or exp Infant, Postmature/ or exp Infant, Premature/ or exp Infant, Very Low Birth Weight/ or exp Infant/ or exp Infant Nutrition Disorders/ or exp Infant, Premature, Diseases/
3. newborn$.mp. or exp Infant, Newborn/
4. baby$.mp.
5. exp Infant, Premature, Diseases/ or exp Infant, Premature/ or exp Premature Birth/ or Preterm$.mp.
6. Prematur$.mp.
7. Postmature&.mp. or exp Infant, Postmature/
8. neonate$.mp.
9. exp Perinatal Care/ or perinat$.mp.
10. postnatal$.mp. or exp Postnatal Care/
11. new?born.mp.
12. or/1-11
13. hypoglyc$.mp. or exp Hypoglycemia/
14. (((low adj sugar) or low) adj glucose).mp.
15. exp Blood Glucose/ or glucose.mp. or exp Glucose/
16. ((low adj sugar) or low adj glucose).mp.
17. 13 or 14 or 15 or 16
18. guideline$.mp.
19. Practice Guideline$.mp. or exp Practice Guideline/
20. Health Planning Guidelines.mp. or exp Health Planning Guidelines/
21. Clinical Protocols.mp. or exp Clinical Protocols/
22. Consensus Development Conference.mp. or exp Consensus Development Conference/
23. Consensus.mp. or Consensus/ or exp Consensus Development Conference, NIH/ or exp Consensus Development Conferences as Topic/
24. Standard of Care.mp. or exp "Standard of Care"/
25. exp Practice Guidelines as Topic/ or Recommendation$s.mp.
26. Clinical Practice Guideline.mp.
27. or/18-26
28. 12 and 17 and 27
29. exp animals/ not humans.sh.
30. 28 not 29
31. limit 30 to yr="2000 - 2022"
